# Supplementary material for: Identification of the Kinase-Substrate Recognition Interface between MYPT1 and Rho-Kinase
Source: Biomolecules. 2022 Jan 18;12(2):159. doi: 10.3390/biom12020159 (PMC8869655; doi:10.3390/biom12020159)
Supplement: Supplementary file 1 [file biomolecules-12-00159-s001.zip › biomolecules-1511000_suppl_R2.5/biomolecules-1511000_suppl_R2.5.pdf]

Supplementary Figures

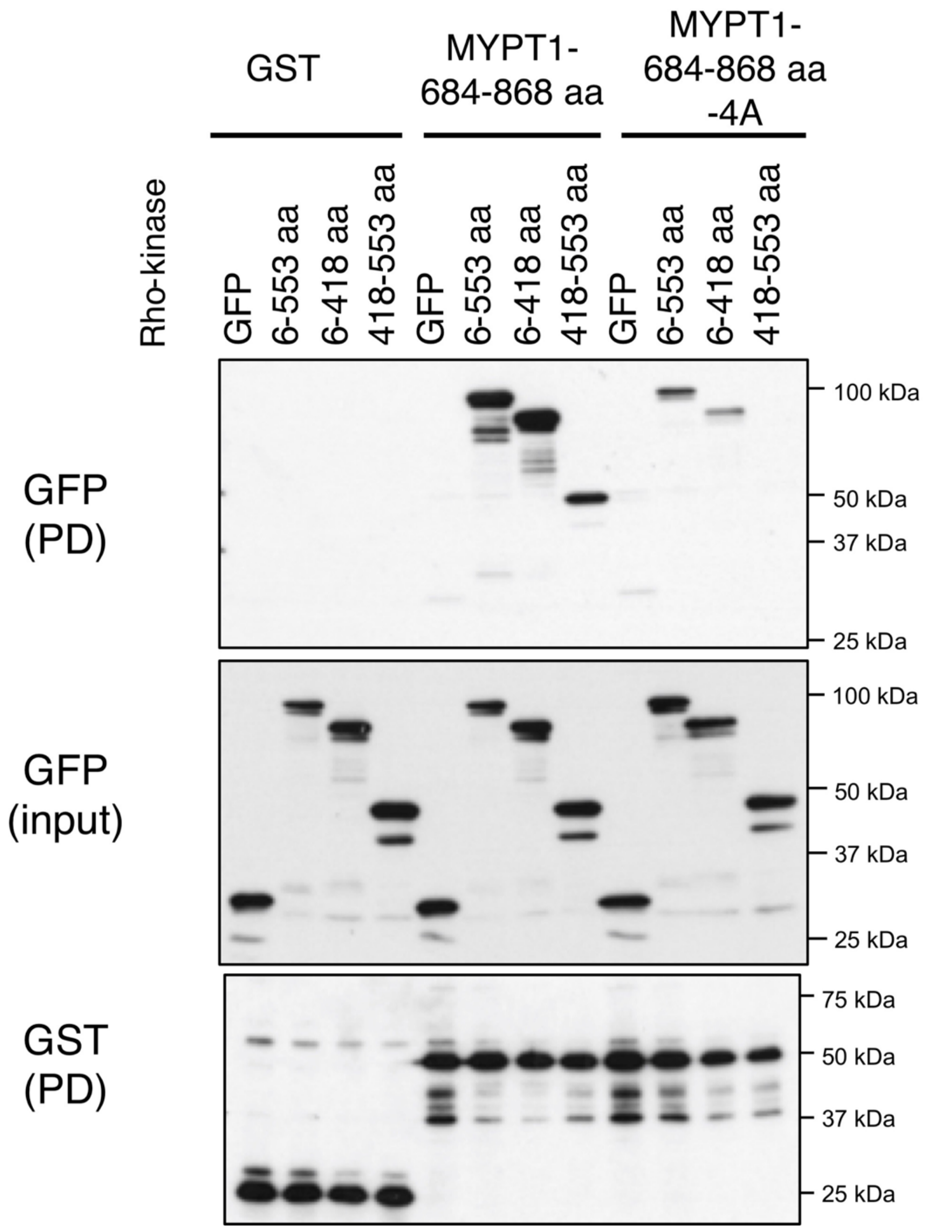

Supplementary Figure S1. Mapping of the MYPT1-interacting region of Rho-kinase-cat.

COS7 cells were cotransfected with the GST-MYPT1 fragment and GFP-Rho-kinase fragment and pulled-down with glutathione beads. The bound proteins were subjected to immunoblot analysis using an anti-GST or anti-GFP antibody. The catalytic domain of Rho-kinase interacted with MYPT1. These results are representative of at least three independent experiments.

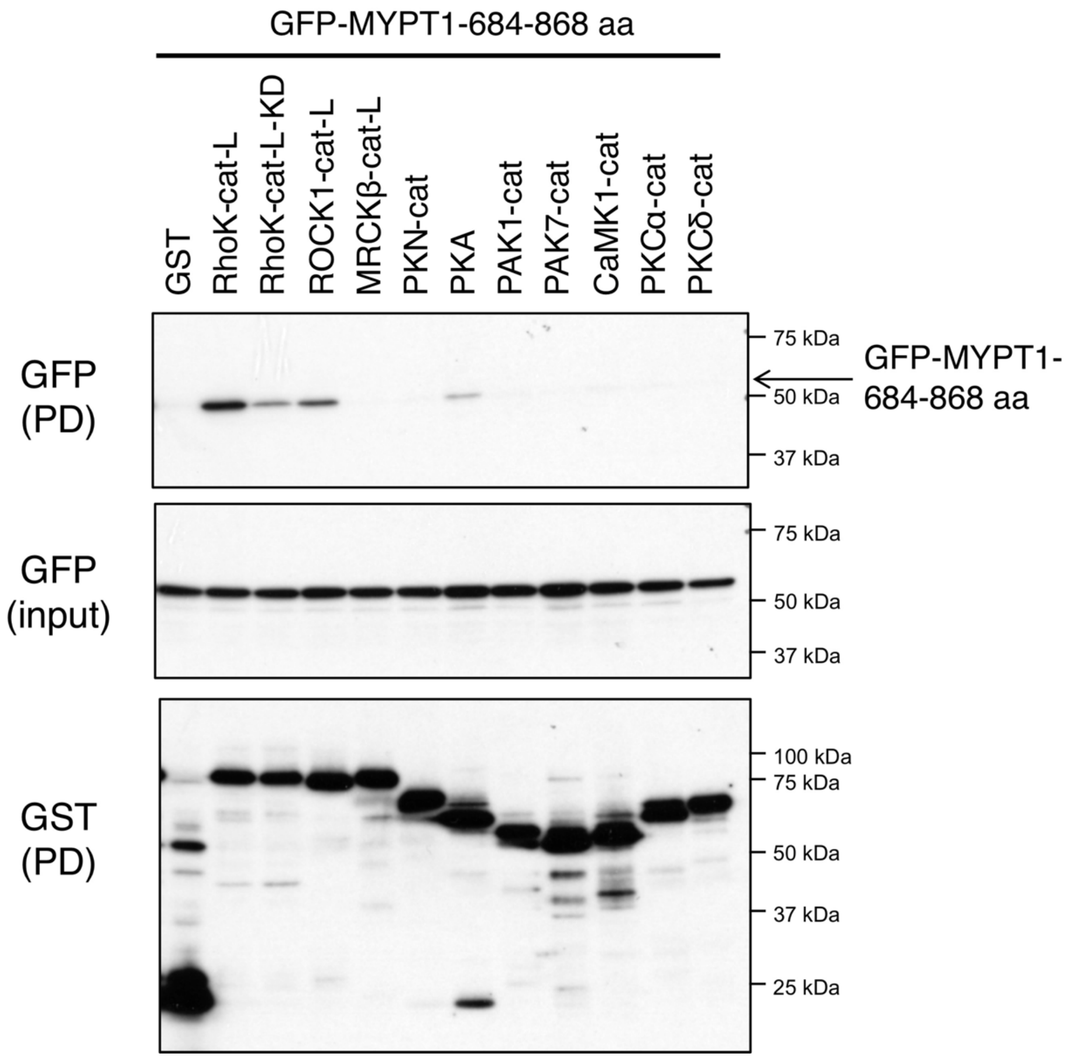

Supplementary Figure S2. Specific binding of Rho-kinase/ROCK to MYPT1.

COS7 cells were cotransfected with the GST-catalytic domain of various kinases and the GFP-MYPT1-684-868 aa fragment and pulled-down with glutathione beads. The bound proteins were subjected to immunoblot analysis using an anti-GST or anti-GFP antibody. These results are representative of at least three independent experiments. Rho-kinase-cat-L-KD; kinase-deficient mutant (K121G) of Rho-kinase-cat-L.

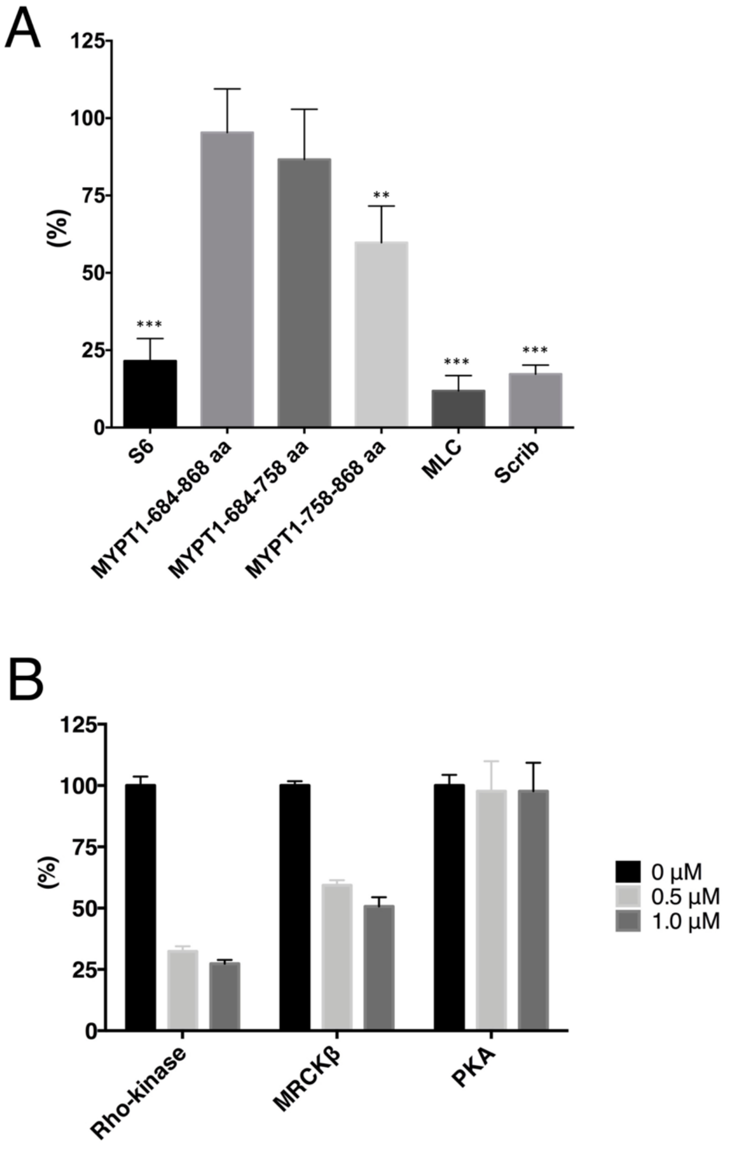

Supplementary Figure S3. Inhibition of the kinase activity by the MYPT1-684-868 aa-4A fragment.

(A) *In vitro* phosphorylation of various substrates by Rho-kinase. S6 rsk substrate peptide (40 μM), GST-MYPT1 fragments (0.5 μM), GST-MLC (0.5 μM), or GST-Scrib-C (1235-1630 aa) (0.5 μM) was phosphorylated by GST-Rho-kinase-cat-L with 1 μM GST or GST-MYPT1-684-868 aa-4A in the presence of γ-[^{32}P]ATP. Data represent means ± SD. N=3. (B) GST-Rho-kinase-cat-L, GST-MRCKβ-cat-L, and GST-PKACα activities were examined using the S6 rsk substrate peptide (40 μM) as a substrate in the presence of γ-[^{32}P]ATP and GST-MYPT1-684-868 aa-4A fragment at the indicated concentrations. The reaction mixtures were applied onto cation-exchange membranes, followed by scintillation counting. Data represent means ± SD. N=3.

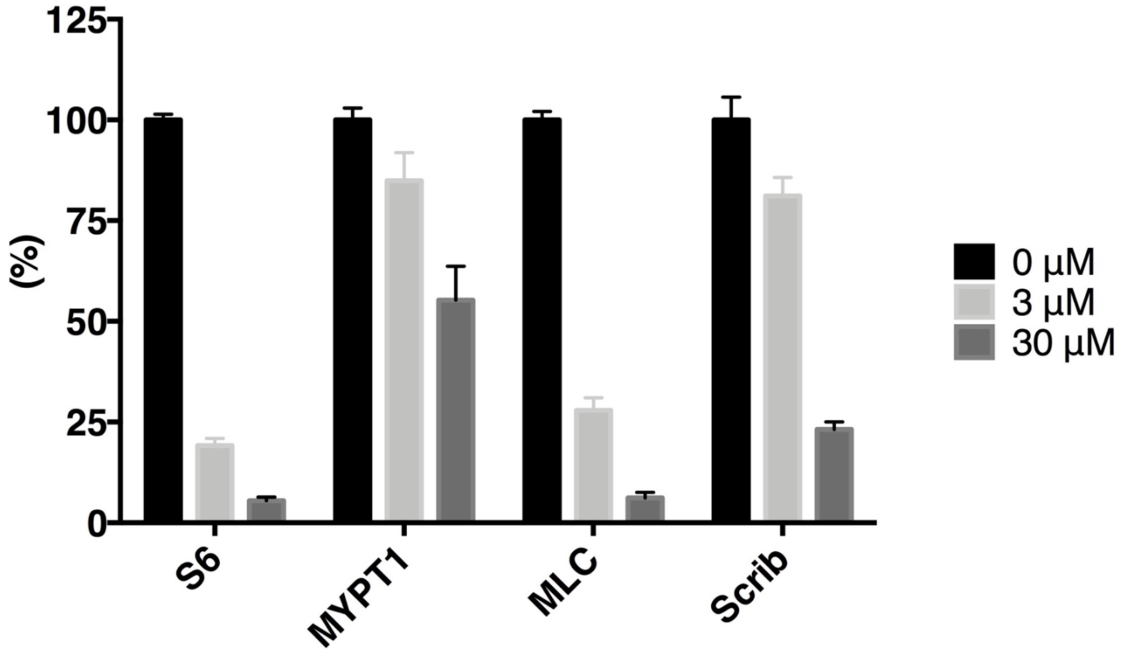

Supplementary Figure S4. Inhibition of the Rho-kinase activity by PS2+DM2 synthetic peptide.

GST-Rho-kinase-cat-L activity was examined using the S6 rsk substrate peptide (40 μM), GST-MYPT1-684-868 aa (0.5 μM), GST-MLC (1 μM), and GST-Scrib-C (1235-1630 aa) (1 μM) as substrates in the presence of γ-[^{32}P]ATP. Synthetic PS2+DM2 peptide was added at the indicated concentrations. The reaction mixtures were applied onto cation-exchange membranes, followed by scintillation counting. Data represent means ± SD. N=3.

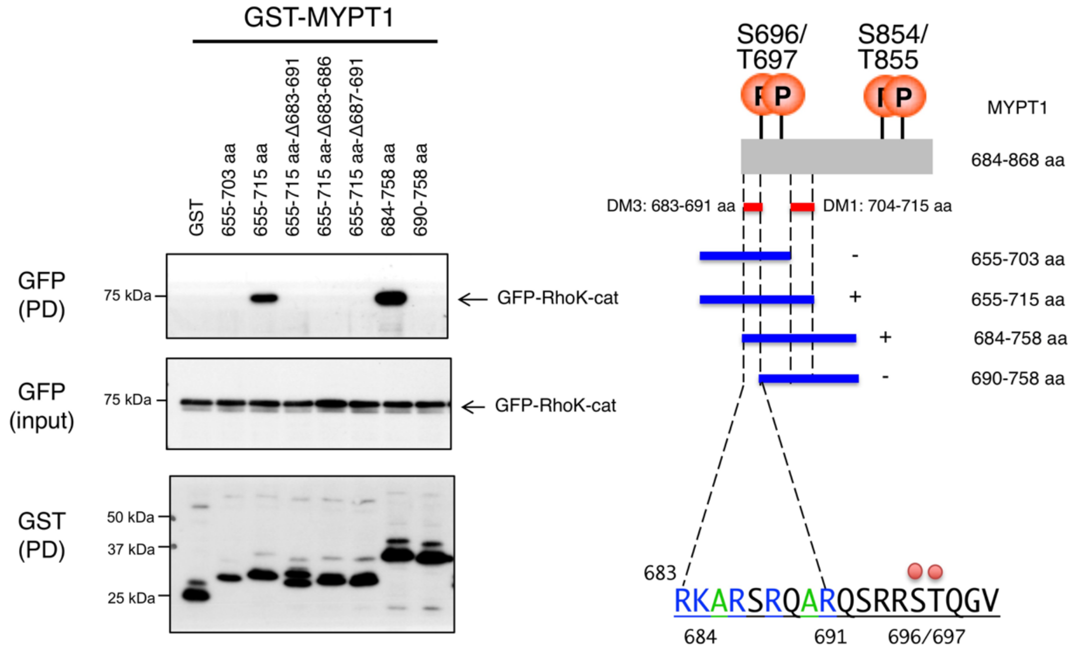

Supplementary Figure S5. Identification of DM3 of MYPT1.

COS7 cells were cotransfected with the GST-MYPT1 fragment and GFP-Rho-kinase-cat and pulled-down with glutathione beads. The bound proteins were subjected to immunoblot analysis using an anti-GST or anti-GFP antibody. The deletion of 683-691 aa (DM3) diminished the association with Rho-kinase. These results are representative of at least three independent experiments.

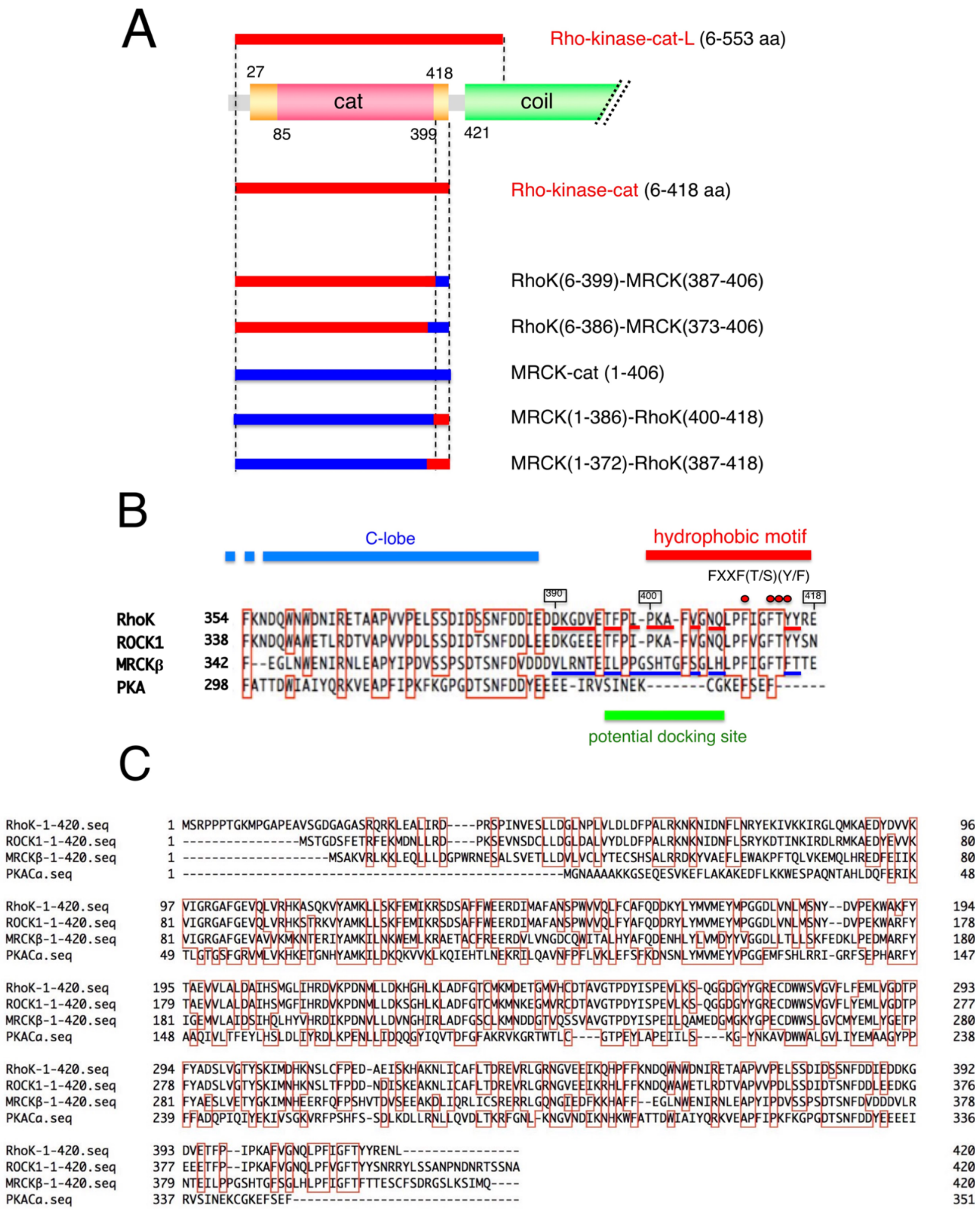

Supplementary Figure S6.

(A) Schematic representation of the domain structure and constructs of Rho-kinase. The core catalytic domain, the N- and C-terminal extensions, and the coiled-coil domain are illustrated in red, orange, and green, respectively. (B) and (C) Sequence alignments of Rho-kinase and related kinases. The C-terminus of the C-lobe and hydrophobic motif in the C-terminal extension of Rho-kinase were compared with ROCK1, MRCKβ, and PKA. Mutated amino acids in Fig. S7B and potential docking sites are indicated.

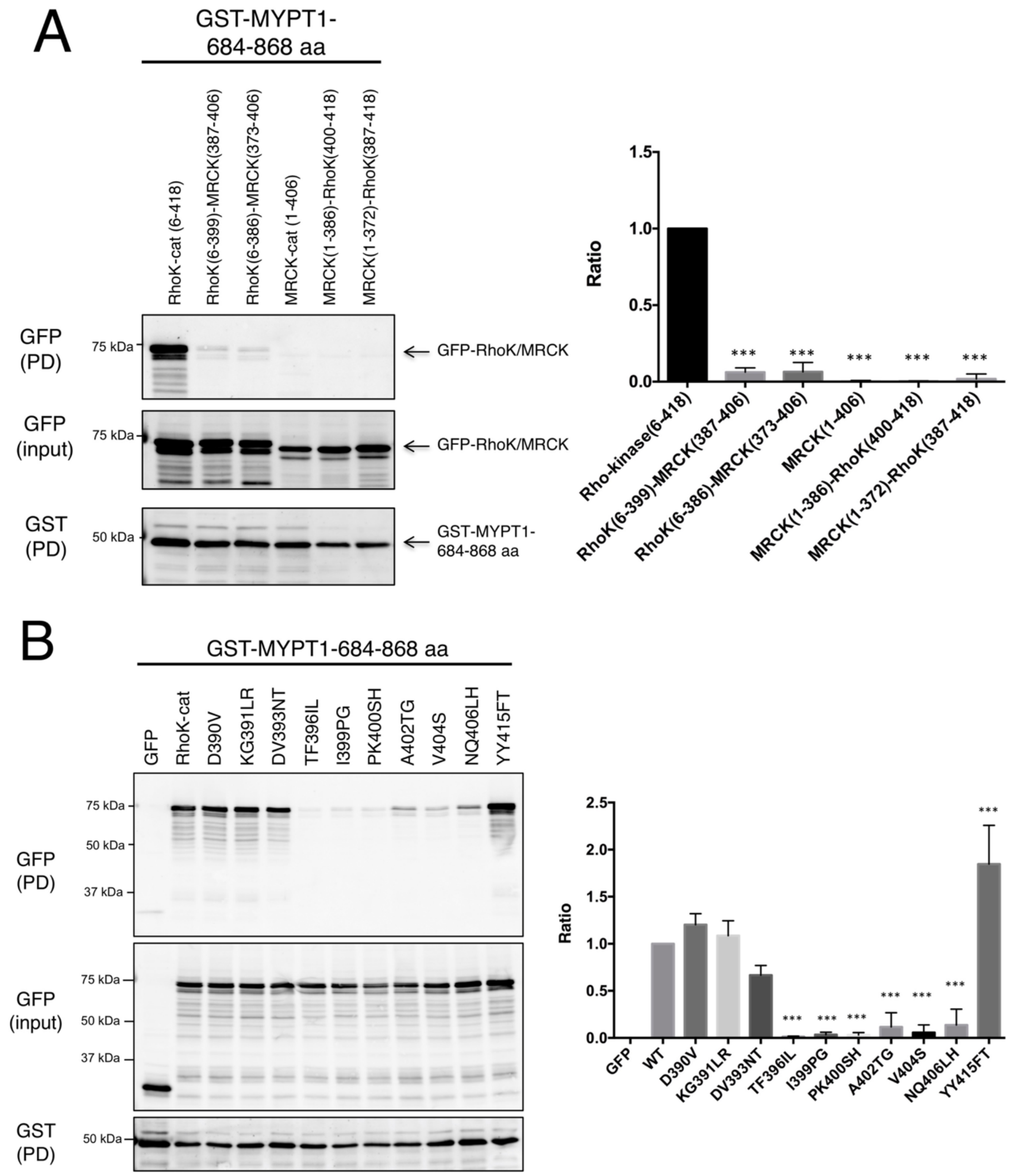

Supplementary Figure S7. Kinase-substrate interface of Rho-kinase.

(A) and (B) Interaction of Rho-kinase-MRCKβ chimera with MYPT1. COS7 cells were cotransfected with the GST-MYPT1-684-868 aa fragment and GFP-Rho-kinase-cat/MRCKβ-cat chimeras and pulled-down with glutathione beads. The bound proteins were subjected to immunoblot analysis using an anti-GST or anti-GFP antibody. Data represent means ± SD. ***, $P < 0.001$ as compared with control.

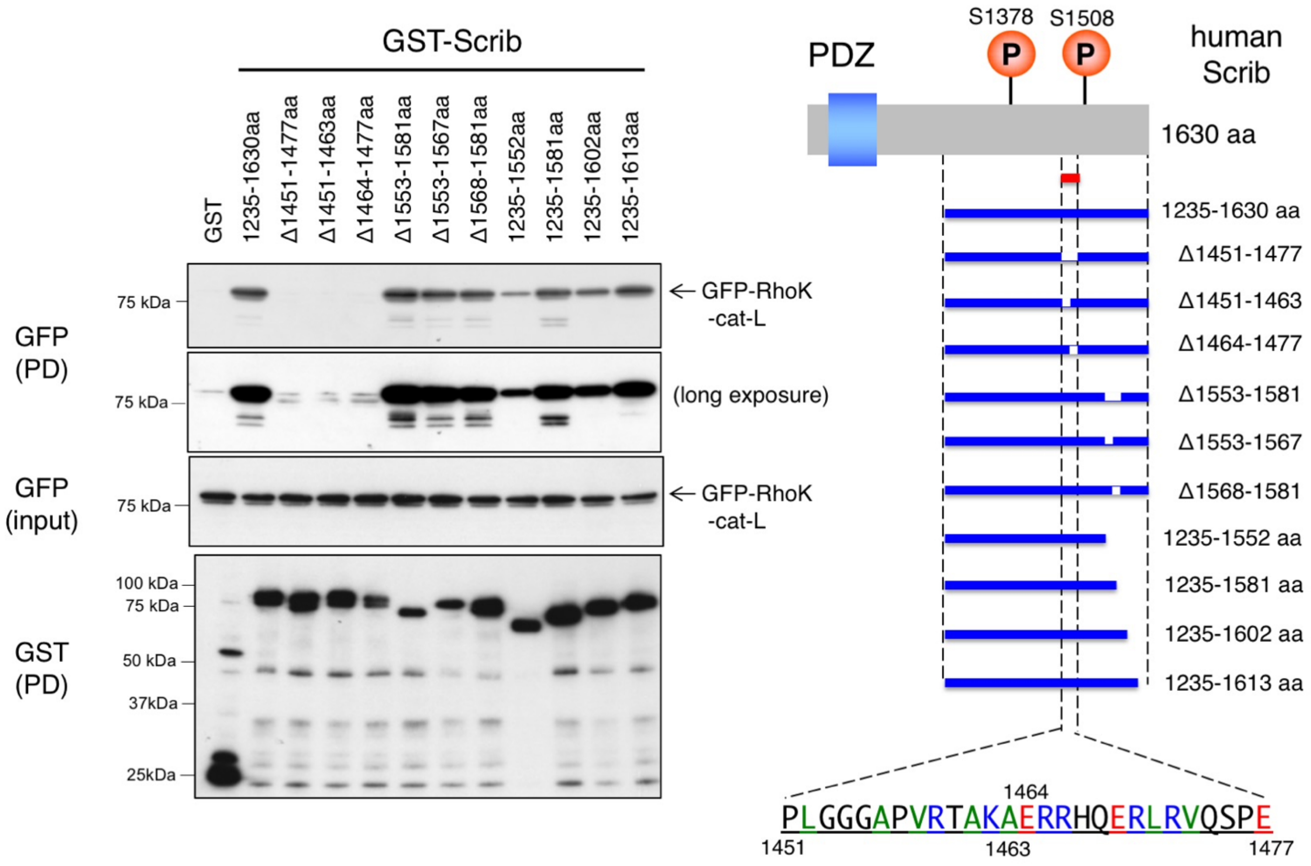

Supplementary Figure S8. Identification of docking motif of Scrib for Rho-kinase.

COS7 cells were cotransfected with the GST-Scrib fragment and GFP-Rho-kinase-cat-L and pulled-down with glutathione beads. The bound proteins were subjected to immunoblot analysis using an anti-GST or anti-GFP antibody. The deletion of 1451-1477 aa diminished the association with Rho-kinase. These results are representative of at least three independent experiments.

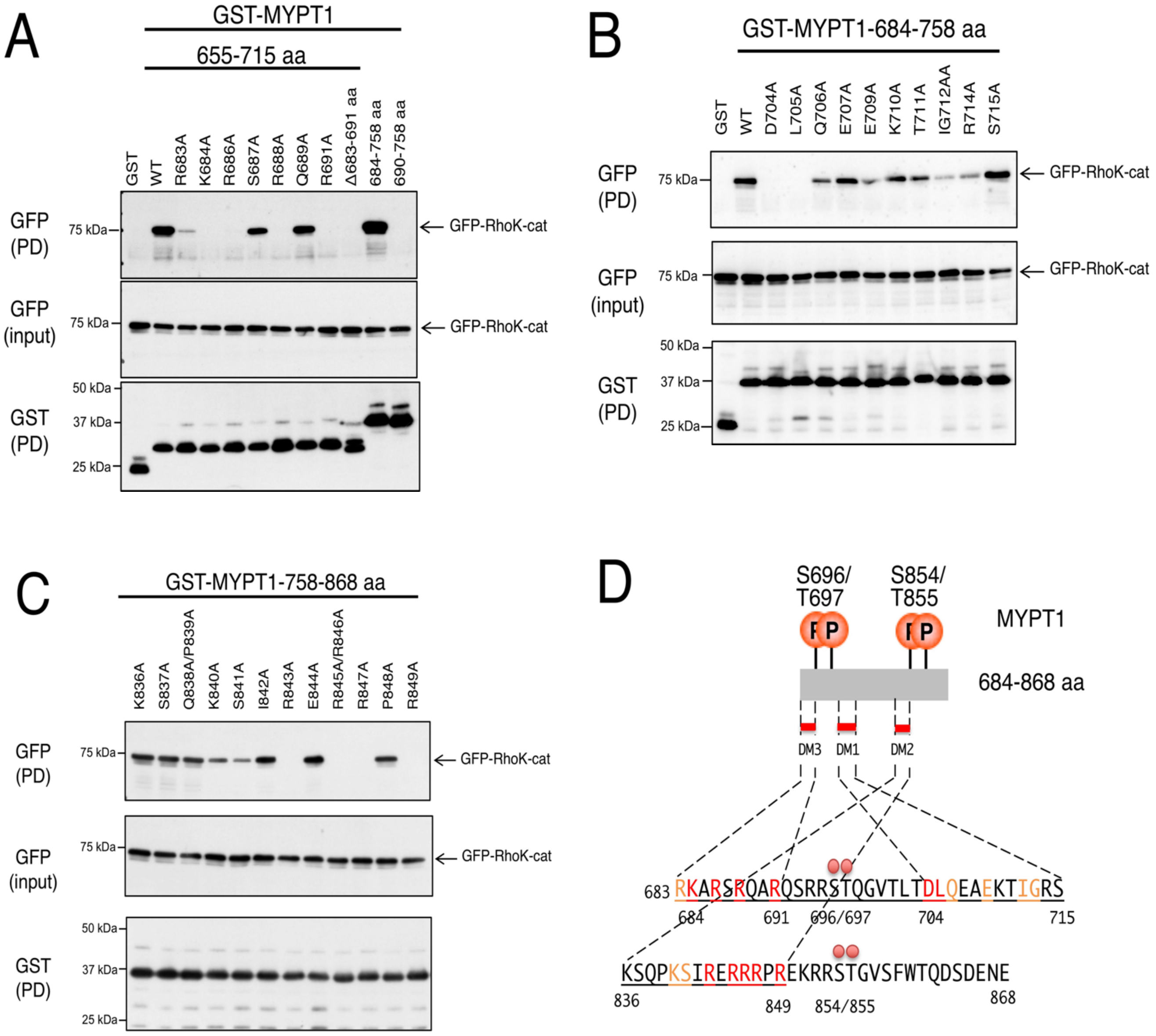

Supplementary Figure S9. Effect of amino acid substitution within DMs of MYPT1 on the binding to Rho-kinase.

(A)-(C) Amino acid within DM3 (A), DM1 (B) and DM2 (C) were replaced by alanine as indicated. COS7 cells were cotransfected with the GST-MYPT1 fragment with mutation and GFP-Rho-kinase-cat and pulled-down with glutathione beads. The bound proteins were subjected to immunoblot analysis using an anti-GST or anti-GFP antibody. These results are representative of three independent experiments. (D) Summary of alanine-scanning. Amino acids demonstrating the strong and mild effects on the binding to Rho-kinase by alanine-scanning are indicated in red and orange, respectively.

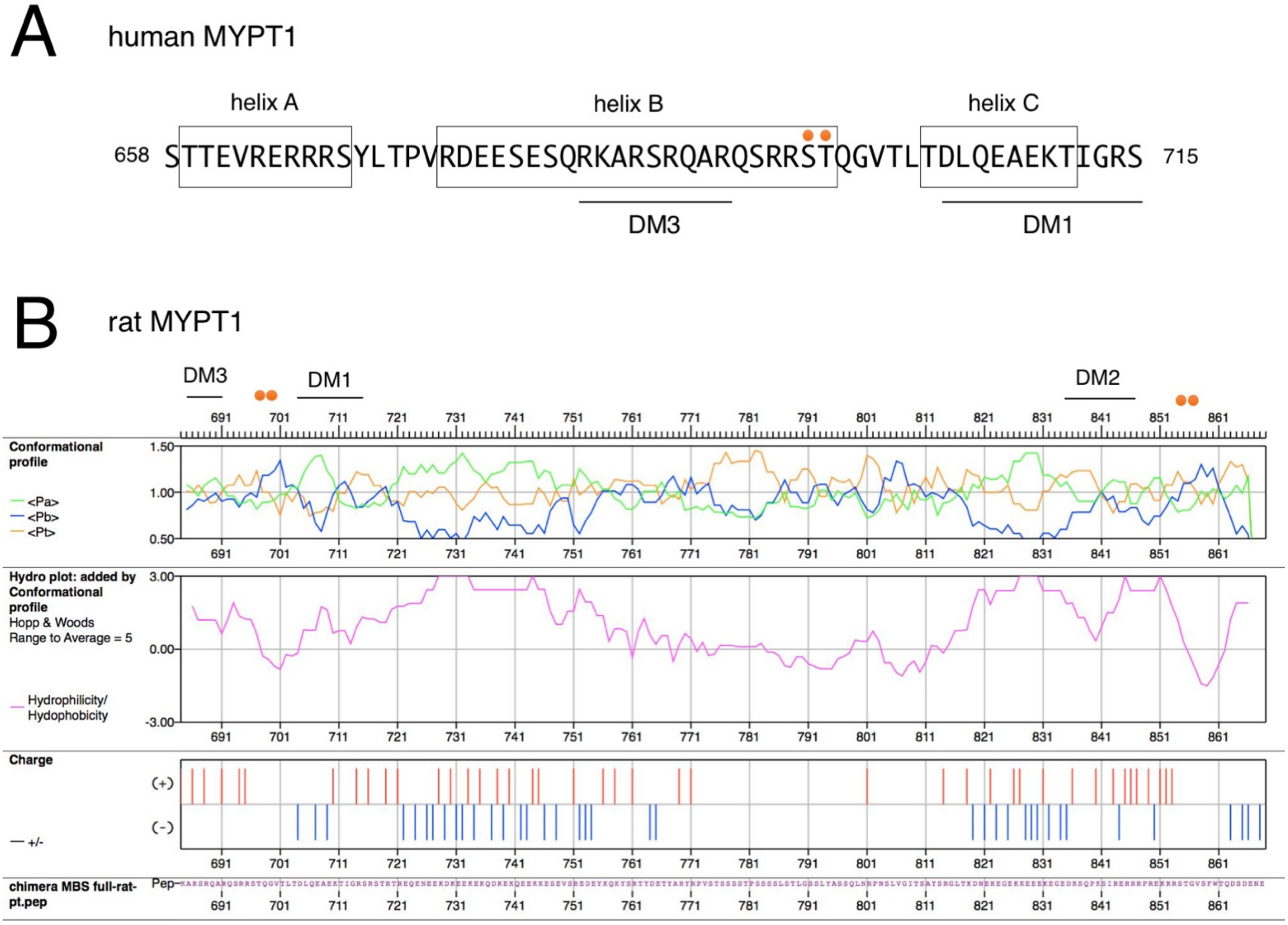

Supplementary Figure S10. Structural information of Rho-kinase-binding region of MYPT1.

(A) Amino acid sequence of human MYPT1 658-714 aa and helical regions reported in Ref [25]. DM1, DM3 and phosphorylation sites (red circles) are indicated. (B) Prediction of secondary structure and hydropathy, and amino acid charge information for rat MYPT1-684-868 aa region were analyzed using GENETYX-MAC software.

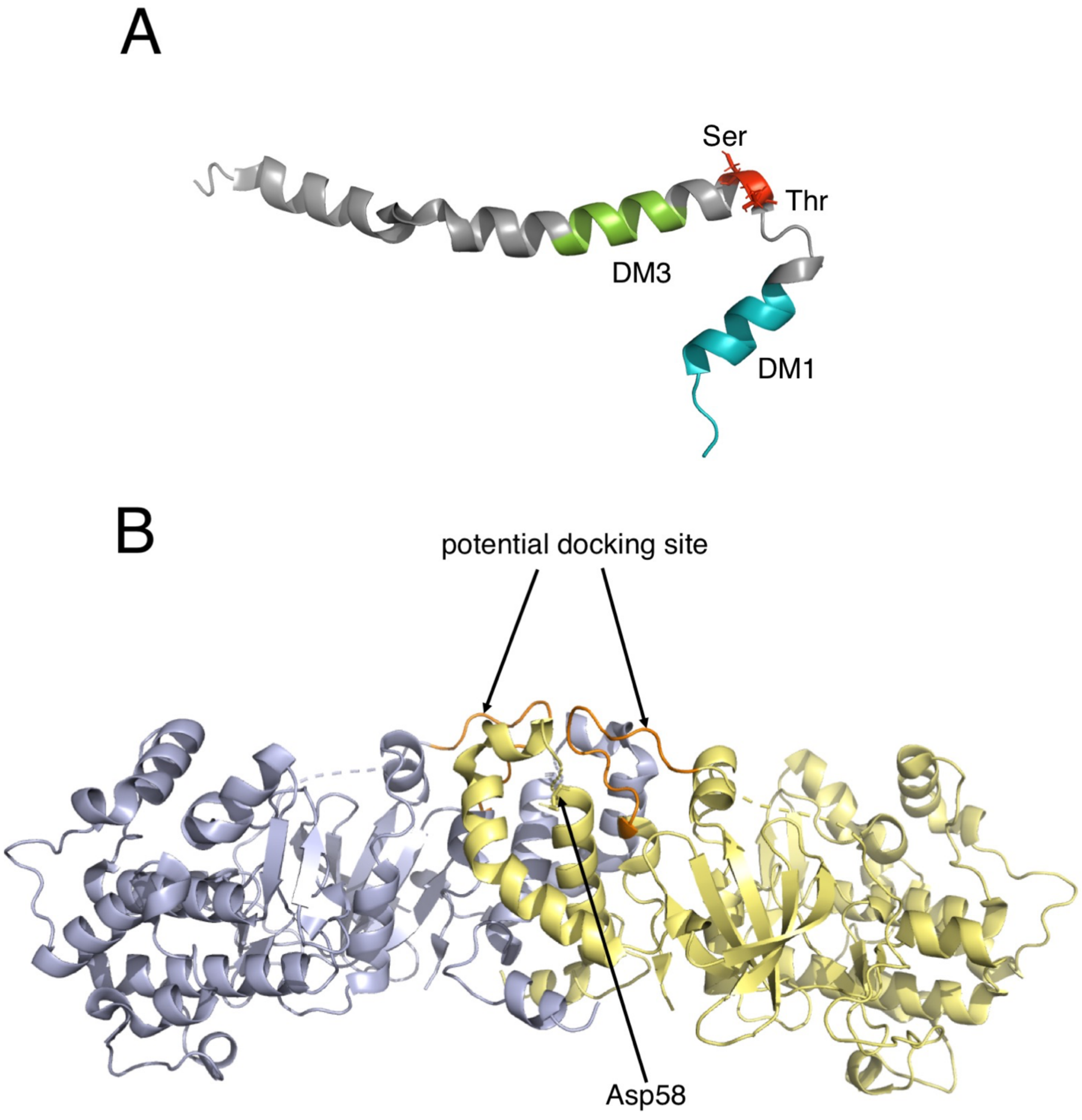

Supplementary Figure S11. Structures of the potential MYPT1-Rho-kinase interfaces.

(A) Positions of DM1 (blue) and DM3 (green) are shown on the ribbon diagram of human MYPT1 658-714 aa (PDB code 2kjy). Phosphorylation sites are indicated in red. (B) Positions of potential docking sites are shown in orange on the ribbon diagram of bovine Rho-kinase-cat homodimer (PDB code 2f2u).

Supplemental Table 1. Kinase activities of Rho-kinase mutants

Rho-kinase mutant	MYPT1 binding	activity
WT	+	100%
D390V	+	124±24%
KG391LR	+	111±12%
DV393NT	+	85±13%
TF396IL	-	24±9%
I399PG	-	24±10%
PK400SH	-	40±11%
A402TG	-	65±13%
V404S	-	33±4%
NQ406LH	-	6±2%
NQ406L	+	95±9%
